# Visit-to-visit HbA1c variability is inversely related to baroreflex sensitivity independently of HbA1c value in type 2 diabetes

**DOI:** 10.1186/s12933-018-0743-7

**Published:** 2018-07-10

**Authors:** Daisuke Matsutani, Masaya Sakamoto, Soichiro Minato, Yosuke Kayama, Norihiko Takeda, Ryuzo Horiuchi, Kazunori Utsunomiya

**Affiliations:** 1 Division of Diabetes, Metabolism and Endocrinology, Department of Internal Medicine, Jikei University School of Medicine, 3-25-8, Nishi-Shinbashi, Minato-ku, Tokyo, 105-8461 Japan; 2 Department of Cardiology, Jikei University School of Medicine, 3-25-8, Nishi-Shinbashi, Minato-ku, Tokyo, 105-8461 Japan; 3 Department of Cardiovascular Medicine, Graduate School of Medicine, The University of Tokyo, 7-3-1, Hongo, Bunkyo-ku, Tokyo, 113-8654 Japan; 4 Department of Pathology, Tsuruoka Kyoritsu Hospital, 9-34, Fumizonomachi, Tsuruoka-shi, Yamagata, 997-0816 Japan

**Keywords:** Visit-to-visit glycemic variability, Long-term glycemic variability, Short-term glycemic variability, Baroreflex sensitivity, Cardiovascular autonomic neuropathy, Continuous glucose monitoring, Type 2 diabetes mellitus

## Abstract

**Background:**

The relationship between long-term glycemic variability (GV) represented by visit-to-visit HbA1c variability and baroreflex sensitivity (BRS) in type 2 diabetes mellitus (T2DM) has not been clarified by previous literature. The present study is the first to examine the relationships between visit-to-visit HbA1c variability and BRS.

**Methods:**

This retrospective study initially analyzed data on 94 patients with T2DM. Visit-to-visit HbA1c variability was evaluated using the intrapersonal coefficient of variation (CV), standard deviation (SD), and adjusted SD of 8 or more serial measurements of HbA1c during a 2-year period. The BRS was analyzed using the sequence method. Short-term GV was assessed by measuring the glucose CV during 24-h continuous glucose monitoring (CGM). The primary objective was to determine if there was a relationship between visit-to-visit HbA1c variability (HbA1c CV) and BRS. Secondary objectives were to examine the relationship between other variables and BRS and the respective and combined effects of long-term GV (HbA1c CV) and short-term GV (CGM CV) on BRS.

**Results:**

A total of 57 patients (mean age 67.2 ± 7.7 years, mean HbA1c 7.3 ± 1.0%) who met this study’s inclusion criteria were finally analyzed. In the univariate analysis, HbA1c CV (*r* = − 0.354, *p* = 0.007), HbA1c SD (*r* = − 0.384, *p* = 0.003), and adjusted HbA1c SD (*r* = − 0.391, *p* = 0.003) were significantly related to low levels of BRS. Multiple regression analysis showed that HbA1c CV, HbA1c SD, and adjusted HbA1c SD were inversely related to BRS. Furthermore, although the increase in either long-term GV (HbA1c CV) or short-term GV (CGM CV) as determined by 24-h CGM was inversely correlated with BRS, additional reductions in BRS were not shown in participants with both HbA1c CV and CGM CV values above the median.

**Conclusions:**

Visit-to-visit HbA1c variability was inversely related to BRS independently of the mean HbA1c in patients with T2DM. Therefore, visit-to-visit HbA1c variability might be a marker of reduced BRS in T2DM.

## Background

Baroreflex sensitivity (BRS), which is a sensitive indicator of cardiovascular autonomic neuropathy (CAN) in type 2 diabetes mellitus (T2DM) [1, 2], has been found to be associated with cardiovascular events [3–5]. In T2DM, the cause of reduced BRS has not been fully elucidated. Reductions in BRS have been reported to be associated with hyperglycemia [6–8], older age [9, 10], obesity [9, 11], hypertension [9, 10, 12], dyslipidemia [10, 13, 14], and increased heart rate [9, 10]. Chronic hyperglycemia is known to be an important cause of reduced BRS in T2DM, and recently we reported that short-term glycemic variability (GV) determined by continuous glucose monitoring (CGM) was inversely related to BRS independently of blood glucose levels [15]. Short-term GV also was reported to be associated with CAN as measured by means other than BRS, such as heart rate variability (HRV) [16] in T2DM; moreover, in type 1 diabetes this relationship was similar to that in T2DM [17, 18]. Recently, not only short-term GV but also long-term GV represented by visit-to-visit HbA1c variability, which is an independent risk factor for cardiovascular events [19–22], were reported as risk factors for CAN [16]. Furthermore, it was reported that visit-to-visit HbA1c variability was a predictor of new-incident peripheral neuropathy [19], and that visit-to-visit glycated albumin variability was significantly associated with the risk of developing CAN in T2DM [23]. Long-term GV refers to glycemic fluctuations over months to years and is generally described as visit-to-visit variability in either HbA1c or fasting blood glucose in T2DM. However, the relationship between such long-term GV represented by visit-to-visit HbA1c variability and BRS has not been clarified.

The present study is the first to examine the relationships between visit-to-visit HbA1c variability and BRS.

## Methods

### Study participants

**Fig. 1:**
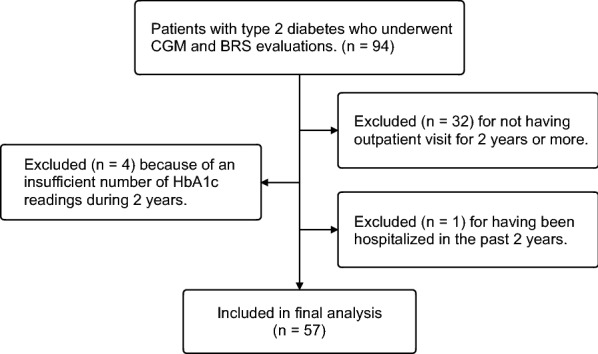
Study population. Fifty-seven participants were analyzed in this study. *CGM* continuing glucose monitoring, *BRS* baroreflex sensitivity

This study retrospectively analyzed data from a previous study on patients whose HbA1c was measured 8 or more times during a 2-year period, including HbA1c values obtained on the first day of BRS measurements [15]. All of the time intervals between HbA1c measurements were within 3 months. The primary objective was to determine if there was a relationship between visit-to-visit HbA1c variability [HbA1c coefficient of variation (CV)] and BRS. Secondary objectives were to examine if there were relationships between BRS and (1) other measurements for evaluating visit-to-visit HbA1c variability [HbA1c standard deviation (SD) and adjusted HbA1c SD]; short-term GV (CGM CV and CGM SD) as determined by CGM; other glycemic control variables such as 2-year mean HbA1c, baseline fasting plasma glucose, and baseline HbA1c level; heart rate; systolic blood pressure (SBP) and diastolic blood pressure (DBP); age; body mass index (BMI); lipid metabolism variables such as triglycerides, low-density lipoprotein (LDL) cholesterol, and high-density lipoprotein (HDL) cholesterol; (2) respective and combined effects of long-term GV (HbA1c CV) and short-term GV (CGM CV) on BRS; and (3) comparison of BRS and visit-to-visit HbA1c variability according to subgroups. The baseline examination was conducted at Jikei University School of Medicine Hospital, Tokyo, Japan and Tsuruoka kyoritsu Hospital, Yamagata, in 2017. Details of inclusion and exclusion criteria were described previously [15]. Briefly, inclusion criteria for that study were age ≥ 40 years and the presence of T2DM diagnosed according to 2017 American Diabetes Association guidelines. Exclusion criteria included arrhythmia, malignancy, and insulin-dependent diabetes mellitus, but did not exclude those with hypertension and dyslipidemia. An additional inclusion criterion in the present study was measurement of HbA1c 8 or more times during a 2-year period. Additionally excluded from the analysis in the current study were patients who had not made an outpatient visit for 2 years or more, had an insufficient number of HbA1c readings during 2 years, and who had been hospitalized due to any disease in the past 2 years (Fig. 1).

Of the 94 people who were finally analyzed for our previous study [15], 57 patients who met this study’s inclusion criteria were finally analyzed after excluding 32 patients who had not made an outpatient visit for 2 years or more, 4 patients with an insufficient number of HbA1c readings during 2 years, and 1 patient who had been hospitalized in the past 2 years (Fig. 1).

### Assessment of visit-to-visit glycemic variability

**Fig. 2:**
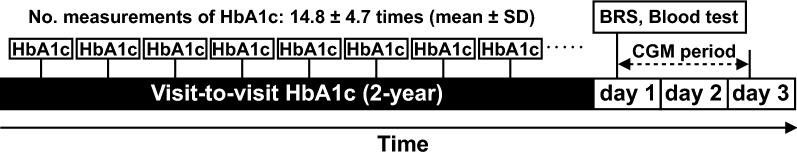
Study protocol. Visit-to-visit HbA1c variability was evaluated using HbA1c values obtained 8 or more times during a 2-year period, including HbA1c values obtained on the first day of measurement of BRS. All of the time intervals between HbA1c measurements were within 3 months. *BRS* baroreflex sensitivity, *CGM* continuous glucose monitoring, *SD* standard deviation

Visit-to-visit HbA1c variability was evaluated using the intrapersonal CV, SD, and adjusted SD of 8 or more serial measurements of HbA1c during a 2-year period, including that obtained on the first day of measuring BRS (Fig. 2). HbA1c was measured 14.8 ± 4.7 times (mean ± SD) during the 2-year period. To adjust for the effect of varying numbers of HbA1c measurements among study patients, the adjusted SD of HbA1c was given as the SD of HbA1c divided by [n/(n − 1)]^0.5^, where n is the number of HbA1c measurements [24].

### Assessment of baroreflex sensitivity

Baroreflex sensitivity was evaluated on the first day of hospitalization in the previous study (Fig. 2) [15]. Using the spontaneous sequence method the beat-to-beat blood pressure (BP) was measured for 15 min after 15 min of supine rest as the slope of the relationship between spontaneous changes in SBP and the pulse interval. Beat-to-beat BP was measured using the second and third fingers of the right hand by the vascular unloading technique. A standard 3-lead electrocardiogram was used to record the heart rate. In calculating BRS, the relative changes in BP (mmHg) and the R–R interval (msec), which is expressed as the distance between corresponding QRS complexes, were determined by the sequence method using cut-off points of 1 mmHg and 3 ms, respectively (Task Force Monitor, CNSystems, Graz, Austria) [25, 26].

### Statistical analyses

Patients’ characteristics and results are presented as mean ± SD or median with interquartile range (IQR) as appropriate according to the data distribution. Pearson’s correlation analysis or Spearman’s rank correlation coefficient test were used for single correlations (Table 2). Multiple-linear regression was used to assess individual and cumulative effects of visit-to-visit HbA1c variability (CV, SD, and adjusted SD), 2-year mean HbA1c, CGM CV, age, sex, BMI, SBP, LDL-cholesterol, and heart rate on BRS. Independent variables were selected based on previous studies of factors associated with low levels of BRS [6–15] (Table 3). As shown in Table 4, individuals were grouped according to CGM CV and HbA1c CV. Group 1 was the reference group and included participants with both CGM CV and HbA1c CV values below the respective median values. Participants in Group 2 had CGM CV values above the median and those in Group 3 had HbA1c CV values above the median. In Group 4 participants had both CGM CV and HbA1c CV values above the median. The analysis of variance (ANOVA) or the Kruskal–Wallis test was used to compare BRS and other variables among the four groups and the Jonckheere trend test was used to test for linear trends in BRS for the four groups. In ANOVA, the Tukey post hoc test or the Games-Howell post hoc test compared results of the BRS and other variables among the four groups. In the Kruskal–Wallis test, the Bonferroni post hoc test compared results of the HbA1c CV among the four groups. As shown in Table 5, HbA1c CV, HbA1c SD, adjusted HbA1c SD, and BRS were divided into the following subgroups: sex, hypertension, dyslipidemia, insulin use, sulfonylurea use, statin use, renin–angiotensin–aldosterone system (RAAS) inhibitor use, calcium-channel blocker use, and beta-blocker use. In subgroup analysis, each parameter was compared using the Student’s *t* test or nonparametric Mann–Whitney *U* test. For data analyses the Statistical Package for the Social Sciences 22.0 software was used (IBM, Armonk, NY, USA). A *p* value < 0.05 was considered significant.

## Results

### Baseline characteristics of study participants

A total of 57 patients were finally analyzed. Baseline clinical and anthropometric characteristics of the study participants are shown in Table 1. The prevalence of study participants ever diagnosed with hypertension or dyslipidemia was 74 or 89%, respectively. The mean age of participants was 67.2 ± 7.7 years, mean duration of diabetes was 11.5 ± 9.6 years, mean number of HbA1c measurements was 14.8 ± 4.7 times, and average of the 2-year mean HbA1c was 7.2 ± 1.0%. Median HbA1c CV was 0.049% (IQR 0.029–0.080%), median HbA1c SD 0.33% (IQR 0.18–0.62%), and median adjusted HbA1c SD was 0.32% (IQR 0.18–0.59%).

**Table 1:** Baseline clinical characteristics of the participants or patients study participants

Variables	
No. patients	57
Sex, male/female	39/18
Age (years)	67.2 ± 7.7
BMI (kg/m^2^)	25.4 ± 4.2
Duration of diabetes (years)	11.5 ± 9.6
Fasting plasma glucose (mg/dL)	134.7 ± 30.7
HbA1c (%)	7.3 ± 1.0
Long-term data	
No. HbA1c measurements in 2 years (times/2 years)	14.8 ± 4.7
Two-year mean HbA1c (%)	7.2 ± 1.0
HbA1c CV (%)	0.049 (0.029–0.080)
HbA1c SD (%)	0.33 (0.18–0.62)
Adjusted HbA1c SD (%)	0.32 (0.18–0.59)
Short-term data	
CGM mean glucose (mg/dL)	154.5 ± 28.8
CGM CV (mg/dL)	23.6 ± 7.1
CGM SD (mg/dL)	36.7 ± 13.2
Hypertension, n (%)	42 (74)
Blood pressure (mmHg)	
Systolic	124.6 ± 17.0
Diastolic	77.1 ± 9.4
Heart rate (beats/min)	69.3 ± 11.8
Dyslipidemia, n (%)	51 (89)
Lipid profile (mg/dL)	
Triglycerides	115 (100–175)
LDL-cholesterol	111.0 ± 30.2
HDL-cholesterol	52.8 ± 15.5
BRS (msec/mmHg)	7.6 ± 2.7

Values are mean ± SD, or median (25th–75th percentiles) or no. (%)

*BMI* body mass index, *SD* standard deviation, *CV* coefficient of variation, *CGM* continuous glucose monitoring, *LDL* low-density lipoprotein, *HDL* high-density lipoprotein, *BRS* baroreflex sensitivity

### Univariate correlates of baroreflex sensitivity

**Fig. 3:**
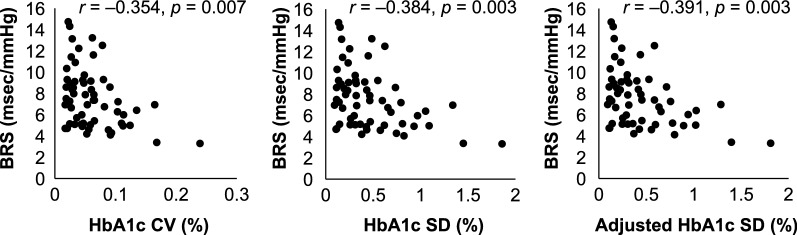
Relationship between visit-to-visit HbA1c variability and baroreflex sensitivity. *BRS* baroreflex sensitivity, *CV* coefficient of variation, *SD* standard deviation

Correlation analysis showed that parameters of visit-to-visit HbA1c variability, such as HbA1c CV (*r* = − 0.354, *p* = 0.007), HbA1c SD (*r* = − 0.384, *p* = 0.003), and adjusted HbA1c SD (*r* = − 0.391, *p* = 0.003), were significantly related to low levels of BRS. In addition to visit-to-visit HbA1c variability, the level of BRS correlated with the 2-year mean HbA1c (*r* = − 0.384, *p* = 0.003), CGM CV (*r* = − 0.325, *p* = 0.014), CGM SD (*r* = − 0.366, *p* = 0.005), heart rate (*r* = − 0.446, *p* = 0.001), and age (*r* = − 0.358, *p* = 0.006) (Table 2, Fig. 3).

**Table 2:** Univariate correlates of baroreflex sensitivity

Variables	*r*	*p*
Fasting plasma glucose (mg/dL)	− 0.173	0.199
HbA1c (%)	− 0.337	0.010
Long-term data		
Two-year mean HbA1c (%)	− 0.384	0.003
HbA1c CV (%)	− 0.354	0.007
HbA1c SD (%)	− 0.384	0.003
Adjusted HbA1c SD (%)	− 0.391	0.003
Short-term data		
CGM mean glucose (mg/dL)	− 0.238	0.074
CGM CV (mg/dL)	− 0.325	0.014
CGM SD (mg/dL)	− 0.366	0.005
Heart rate (beats/min)	− 0.446	0.001
SBP (mmHg)	0.154	0.252
DBP (mmHg)	0.092	0.498
Age (years)	− 0.358	0.006
BMI (kg/m^2^)	0.006	0.965
Triglycerides (mg/dL)	0.085	0.527
LDL-cholesterol (mg/dL)	0.074	0.586
HDL-cholesterol (mg/dL)	− 0.088	0.514

*CV* coefficient of variation, *SD* standard deviation, *CGM* continuous glucose monitoring, *SBP* systolic blood pressure, *DBP* diastolic blood pressure, *BMI* body mass index, *LDL* low-density lipoprotein, *HDL* high-density lipoprotein

### Multiple regression analysis of baroreflex sensitivity

Multiple regression analysis showed that HbA1c CV, HbA1c SD, and adjusted HbA1c SD were inversely related to BRS. These findings remained after adjusting BRS for the 2-year mean HbA1c, CGM CV, age, sex, BMI, SBP, LDL-cholesterol, and heart rate. In addition to parameters of visit-to-visit HbA1c variability, age, CGM CV, and heart rate were found to be predictive factors for BRS (Table 3).

**Table 3:** Multiple regression analysis of baroreflex sensitivity

Independent variables	Univariate		Multivariate					
	*r*	*p*	Model 1		Model 2		Model 3	
			*β*	*p*	*β*	*P*	*β*	*p*
(a)								
HbA1c CV (%)	− 0.354	0.007	− 0.341	0.020	− 0.359	0.014	− 0.339	0.014
Two-year mean HbA1c (%)	− 0.384	0.003	− 0.073	0.641	− 0.052	0.741	− 0.006	0.969
CGM CV (mg/dL)	− 0.325	0.014	− 0.288	0.035	− 0.308	0.025	− 0.229	0.072
Age (years)	− 0.358	0.006	− 0.323	0.008	− 0.330	0.006	− 0.302	0.008
Sex (male/female)	–	0.550	− 0.129	0.283	− 0.161	0.180	− 0.149	0.182
BMI (kg/m^2^)	0.006	0.965	− 0.221	0.120	− 0.205	0.127	− 0.159	0.212
SBP (mmHg)	0.154	0.252	0.067	0.585	–	–	–	–
LDL-cholesterol (mg/dL)	0.074	0.586	–	–	0.122	0.297	–	–
Heart rate (beats/min)	− 0.446	0.001	–	–	–	–	− 0.300	0.011
(b)								
HbA1c SD (%)	− 0.384	0.003	− 0.373	0.021	− 0.395	0.014	− 0.371	0.014
Two-year mean HbA1c (%)	− 0.384	0.003	− 0.020	0.906	0.006	0.971	0.048	0.769
CGM CV (mg/dL)	− 0.325	0.014	− 0.288	0.035	− 0.309	0.025	− 0.230	0.071
Age (years)	− 0.358	0.006	− 0.322	0.008	− 0.328	0.006	− 0.301	0.008
Sex (male/female)	–	0.550	− 0.130	0.278	− 0.163	0.176	− 0.150	0.179
BMI (kg/m^2^)	0.006	0.965	− 0.228	0.111	− 0.215	0.113	− 0.167	0.192
SBP (mmHg)	0.154	0.252	0.064	0.606	–	–	–	–
LDL-cholesterol (mg/dL)	0.074	0.586	–	–	0.123	0.290	–	–
Heart rate (beats/min)	− 0.446	0.001	–	–	–	–	− 0.299	0.011
(c)								
Adjusted HbA1c SD (%)	− 0.391	0.003	− 0.376	0.020	− 0.397	0.014	− 0.376	0.013
Two-year mean HbA1c (%)	− 0.384	0.003	− 0.017	0.919	0.007	0.967	0.051	0.752
CGM CV (mg/dL)	− 0.325	0.014	− 0.290	0.034	− 0.309	0.025	− 0.231	0.070
Age (years)	− 0.358	0.006	− 0.318	0.009	− 0.325	0.007	− 0.297	0.009
Sex (male/female)	–	0.550	− 0.131	0.273	− 0.163	0.173	− 0.152	0.174
BMI (kg/m^2^)	0.006	0.965	− 0.227	0.111	− 0.213	0.115	− 0.166	0.193
SBP (mmHg)	0.154	0.252	0.065	0.596	–	–	–	–
LDL-cholesterol (mg/dL)	0.074	0.586	–	–	0.122	0.297	–	–
Heart rate (beats/min)	− 0.446	0.001	–	–	–	–	− 0.301	0.010

Dependent variable was baroreflex sensitivity, and the independent variables were Model 1, Model 2, and Model 3. Model 1: age, sex, BMI, CGM CV, mean HbA1c, visit-to-visit HbA1c variability, and SBP; Model 2: age, sex, BMI, CGM CV, mean HbA1c, visit-to-visit HbA1c variability, and LDL-cholesterol; Model 3: age, sex, BMI, CGM CV, mean HbA1c, visit-to-visit HbA1c variability, and heart rate; Visit-to-visit HbA1c variability was (a) HbA1c CV (b) HbA1c SD, and (c) adjusted HbA1c SD; Model 1 (a) R-squared 0.398, adjusted R-squared 0.312, (b) R-squared 0.398, adjusted R-squared 0.312, (c) R-squared 0.399, adjusted R-squared 0.313; Model 2 (a) R-squared 0.408, adjusted R-squared 0.324, (b) R-squared 0.409, adjusted R-squared 0.324, (c) R-squared 0.409, adjusted R-squared 0.325; Model 3 (a) R-squared 0.471, adjusted R-squared 0.395, (b) R-squared 0.471, adjusted R-squared 0.395, (c) R-squared 0.472, adjusted R-squared 0.397

*CV* coefficient of variation, *SD* standard deviation, *CGM* continuous glucose monitoring, *BMI* body mass index, *SBP* systolic blood pressure, *LDL* low-density lipoprotein

### Respective and combined effects of short-term and long-term glycemic variability on baroreflex sensitivity

**Fig. 4:**
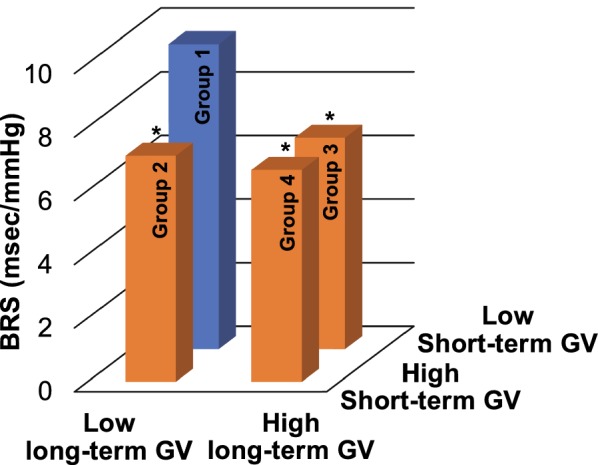
Respective and combined effects of long-term (HbA1c CV) and short-term (CGM CV) glycemic variability on baroreflex sensitivity. *BRS* baroreflex sensitivity, *GV* glycemic variability, *CGM* continuous glucose monitoring, *CV* coefficient of variation. *p < 0.05 vs. Group 1

Table 4 shows comparisons of BRS among the four groups based on ANOVA. Group 1 included participants with both CGM CV and HbA1c CV values below the respective median values while Group 2 included only participants with CGM CV values above the median and Group 3 included only participants with HbA1c CV values above the median. In Group 4 participants had both CGM CV and HbA1c CV values above the median. There was a significant difference in BRS among these four groups (*p* = 0.004). The results were then analyzed by the Tukey post hoc test. Group 2 (*p* = 0.045), Group 3 (*p* = 0.012), and Group 4 (*p* = 0.009) had reduced BRS in comparison with Group 1 (Table 4, Fig. 4). However, Group 4 did not have reduced BRS in comparison with Group 2 (*p* = 0.963) and Group 3 (*p* = 1.000). This observation was confirmed by the Jonckheere trend test: BRS (*p* = 0.002) showed a significant decreasing trend from Group 1 to Group 4.

**Table 4:** Respective and combined effects of short-term and long-term glycemic variability on baroreflex sensitivity

Variables	Group 1 (n = 16)	Group 2 (n = 13)	Group 3 (n = 13)	Group 4 (n = 15)	*p* value^§^	Test for trend *p* value
BRS (msec/mmHg)						
Mean ± SD	9.58 ± 3.0	7.10 ± 1.9*	6.64 ± 2.2*	6.66 ± 2.4*	0.004	0.002
*p* value		0.045	0.012	0.009		
Age (years)	66.9 ± 5.6	67.2 ± 7.5	65.9 ± 8.4	68.6 ± 9.6	0.840	
Diabetes duration (years)	8.3 ± 8.2	14.3 ± 12.4	8.2 ± 4.2	15.4 ± 10.4	0.069	
CGM CV (mg/dL)	18.3 ± 2.5	29.5 ± 5.5*	18.1 ± 3.5^†^	29.1 ± 5.9*^‡^	0.000	
Two-year mean HbA1c (%)	6.6 ± 0.4	6.8 ± 0.8	7.6 ± 0.8*	7.8 ± 1.1*^†^	0.000	
HbA1c CV (%)	0.030 (0.024–0.044)	0.027 (0.021–0.033)	0.065 (0.060–0.114)*^†^	0.082 (0.064–0.107)*^†^	0.000	

Values are mean ± SD or median (25th–75th percentiles). Group 1, both CGM CV and HbA1c CV below median CV value. Group 2, CGM CV only above median. Group 3, HbA1c CV only above median. Group 4, both CGM CV and HbA1c CV above median values

*BRS* baroreflex sensitivity, *CV* coefficient of variation, *SD* standard deviation, *CGM* continuous glucose monitoring

Results of the Tukey post hoc test, the Games-Howell post hoc test, or the Bonferroni post hoc test (1) compared with Group 1: *p < 0.05; (2) compared with Group 2: ^†^*p *< 0.05; (3) compared with Group 3: ^‡^*p* < 0.05

^§^ The analysis of variance (ANOVA) or the Kruskal–Wallis test was used to compare BRS among the four groups

### Comparison of baroreflex sensitivity in subgroups

Use of sulfonylurea was associated with low levels of BRS compared with its non-use (sulfonylurea use vs. non-use: 6.4 ± 2.1 vs. 8.1 ± 2.8 ms/mmHg, *p* = 0.028). The HbA1c CV, HbA1c SD, and adjusted HbA1c SD in patients taking sulfonylurea were larger than in those who did not (sulfonylurea use vs. non-use: median HbA1c CV 0.065% [IQR 0.035–0.103%] vs. 0.047% [IQR 0.025–0.065%], *p *= 0.043; median HbA1c SD 0.60% [IQR 0.26–0.88%] vs. 0.32% [IQR 0.16–0.47%], *p* = 0.018; median adjusted HbA1c SD 0.59% [IQR 0.25–0.85%] vs. 0.30% [IQR 0.16–0.46%], *p* = 0.015). Hypertension and statin use were associated with low levels of BRS (hypertensive vs. normotensive: 7.0 ± 2.5 vs. 9.1 ± 2.9 ms/mmHg, *p* = 0.011; statin use vs. non-use: 6.6 ± 2.6 vs. 8.1 ± 2.6 ms/mmHg, *p* = 0.035). However, there was no significant relationship between the mean BRS and sex, dyslipidemia, and the use of insulin, RAAS inhibitors, calcium-channel blockers, and beta-blockers (Table 5).

**Table 5:** Comparison of baroreflex sensitivity according to subgroups

Subgroup	No (%)	HbA1c CV (%)	*p* value	HbA1c SD (%)	*p* value	Adjusted HbA1c SD (%)	*p* value	BRS (msec/mmHg)	*p* value
Sex			0.140		0.186		0.181		0.550
Male	39 (68)	0.053 (0.032–0.082)		0.37 (0.23–0.66)		0.36 (0.23–0.64)		7.7 ± 2.7	
Female	18 (32)	0.040 (0.021–0.070)		0.29 (0.13–0.59)		0.27 (0.13–0.57)		7.3 ± 2.8	
Hypertension			0.088		0.107		0.095		0.011
Yes	42 (74)	0.055 (0.032–0.090)		0.40 (0.24–0.67)		0.39 (0.23–0.64)		7.0 ± 2.5*	
No	15 (26)	0.032 (0.026–0.059)		0.21 (0.17–0.62)		0.20 (0.16–0.59)		9.1 ± 2.9	
Dyslipidemia			0.585		0.694		0.713		0.903
Yes	51 (89)	0.049 (0.029–0.089)		0.33 (0.18–0.66)		0.31 (0.18–0.64)		7.6 ± 2.6	
No	6 (11)	0.051 (0.024–0.060)		0.33 (0.15–0.51)		0.32 (0.14–0.49)		7.7 ± 3.7	
Insulin use			0.422		0.233		0.223		0.655
Yes	6 (11)	0.061 (0.042–0.091)		0.54 (0.32–0.83)		0.52 (0.31–0.80)		7.1 ± 3.8	
No	51 (89)	0.048 (0.028–0.082)		0.32 (0.18–0.63)		0.31 (0.17–0.59)		7.6 ± 2.6	
Sulfonylurea use			0.043		0.018		0.015		0.028
Yes	17 (30)	0.065 (0.035–0.103)*		0.60 (0.26–0.88)*		0.59 (0.25–0.85)*		6.4 ± 2.1*	
No	40 (70)	0.047 (0.025–0.065)		0.32 (0.16–0.47)		0.30 (0.16–0.46)		8.1 ± 2.8	
Statin use			0.108		0.132		0.124		0.035
Yes	20 (35)	0.062 (0.041–0.105)		0.45 (0.27–0.80)		0.44 (0.26–0.76)		6.6 ± 2.6*	
No	37 (65)	0.047 (0.027–0.065)		0.32 (0.17–0.51)		0.30 (0.16–0.49)		8.1 ± 2.6	
RAAS inhibitor use			0.635		0.600		0.544		0.526
Yes	22 (39)	0.049 (0.032–0.069)		0.35 (0.24–0.52)		0.34 (0.23–0.50)		7.3 ± 2.6	
No	35 (61)	0.049 (0.027–0.089)		0.32 (0.17–0.63)		0.30 (0.17–0.60)		7.8 ± 2.8	
CCB use			0.272		0.349		0.324		0.241
Yes	24 (42)	0.062 (0.030–0.087)		0.44 (0.24–0.65)		0.43 (0.23–0.63)		7.1 ± 2.4	
No	33 (58)	0.045 (0.028–0.080)		0.31 (0.18–0.63)		0.30 (0.17–0.59)		7.9 ± 2.9	
Beta-blocker use			0.902		1.000		0.989		0.462
Yes	5 (9)	0.048 (0.027–0.085)		0.33 (0.20–0.71)		0.32 (0.19–0.69)		8.4 ± 3.7	
No	52 (91)	0.050 (0.028–0.081)		0.33 (0.18–0.63)		0.31 (0.17–0.60)		7.5 ± 2.6	

Values are mean ± SD, median (25th–75th percentiles) or no. (%)

*BRS* baroreflex sensitivity, *CV* coefficient of variation, *SD* standard deviation, *RAAS* renin–angiotensin–aldosterone system, *CCB* calcium-channel blocker

* *p* value corresponds to the Student’s *t* test or the non-parametric Mann–Whitney *U*-test

## Discussion

This is the first clinical study to assess the relationship between BRS and long-term GV as represented by visit-to-visit HbA1c variability. We retrospectively assessed data on patients with T2DM whose HbA1c was examined 8 or more times during the 2 years beginning from the time of recruitment for participation in our previous prospective study [15].

The results showed that visit-to-visit HbA1c variability was inversely correlated with BRS (Table 2, Fig. 3). In the multiple regression analysis, visit-to-visit HbA1c variability was independently associated with a decrease in BRS (Table 3). Furthermore, although the increase in either long-term GV or short-term GV was inversely correlated with BRS, an additional reduction in BRS was not shown in participants with both long-term GV and short-term GV values above the median (Table 4, Fig. 4). As in previous reports, our analysis showed that age and heart rate were also independent predictors of BRS [9, 10] (Tables 2, 3).

Long-term GV emerged as another measure of glycemic control that better predicted cardiovascular events [19–22] and microvascular complications [19, 21, 27, 28] than the average HbA1c level. Since there has been more evidence that long-term GV was related to prognosis and microvascular complications than short-term GV [29–34], long-term GV may confirm the prognosis to a greater extent. This is the first clinical study to investigate the association between long-term GV and BRS that can quantitatively and sensitively evaluate CAN [1]. Our results further support existing data showing that there was an independent association of visit-to-visit HbA1c variability with the presence of CAN [16] and that visit-to-visit HbA1c variability was a predictor of new-incident peripheral neuropathy [19]. Also we noted that visit-to-visit glycated albumin variability was significantly associated with the risk of developing CAN in T2DM as previously reported [23].

Several potential mechanisms may link increased GV to the reduced BRS from a pathophysiological point of view. Previous studies suggested that increased GV causes the reduced BRS by inducing endothelial dysfunction and increasing oxidative stress independently of chronic hyperglycemia. For example, GV was shown to induce endothelial dysfunction [35–38], which subsequently causes neuropathy [39–42]. GV increased oxidative stress [36–38, 43] causing neuropathy [44, 45]. In particular, vascular endothelial dysfunction leads to hypoxia and blood flow disorders in neuronal cells [39, 40], which might result in autonomic dysfunction. However, since this phenomenon is difficult to prevent or ameliorate by anti-diabetic drugs, these conditions persist for long periods and the autonomic dysfunction possibly becomes irreversible or worsens. Furthermore, insulin resistance may be one possible explanation of the result showing that increased visit-to-visit HbA1c variability was related to reduced BRS, because GV is known to be associated with insulin resistance [46]. Insulin resistance was shown to be associated with sympathetic activity [47], which is a determinant of BRS [48]. Although it was previously reported that long-term GV was associated with the severity of CAN compared to short-term GV [16], in this study the effects of long-term GV and short-term GV on reduced BRS were comparable. Furthermore, an additional reduction in BRS was not shown in participants with both long-term GV and short-term GV values above the median (Table 4, Fig. 4). Although endothelial function, oxidative stress, and insulin resistance were not examined in this study, an increase in either long-term GV or short-term GV reduces BRS to some extent by these physiological mechanisms and BRS might have reached a steady state in these study participants. In addition, our results may have been due to the fact that the evaluation period for long-term GV of 2 years was insufficient, and the duration of hypertension and the state of its management were not evaluated.

On the other hand, in subgroup analysis, patients taking sulfonylurea had larger visit-to-visit HbA1c variability than those who did not. Furthermore, sulfonylureas were associated with reduced BRS (Table 5). Sulfonylureas are prescribed typically for T2DM in patients with relative difficulty in glycemic control, such as those with a long duration of diabetes and low insulin levels. Furthermore, sulfonylureas present a high risk of causing hypoglycemia [49–51], and as a result visit-to-visit HbA1c variability may have increased in those patients taking sulfonylureas.

In addition to long-term GV, as previously reported [9, 10], because our study showed that age and heart rate were independently correlated with BRS, these factors are important to consider when assessing BRS, especially in elderly patients with T2DM and a high heart rate. It is known that loss of arterial distensibility is the major mechanism responsible for the reduction of BRS in elderly patients [52]. Since the baroreflex modulates the heart rate, the association of BRS with heart rate is not unexpected. A low heart rate indicates high vagal tone, which usually accompanies high BRS [53]. On the other hand, unlike previous reports [9–14], we did not find a significant correlation of BRS with blood pressure, BMI, and lipid metabolism variables. That this study enrolled patients who were taking antihypertensive agents and/or lipid lowering agents that may improve BRS [54–56] might have influenced the results.

This study has four notable limitations. First, in this retrospective study, only 57 patients were enrolled and analyzed. Second, the period studied was short, that is, only 2 years, and changes in anti-diabetic drugs were not considered during the 2-year period. Third, factors related to drugs that could affect BRS, such as anti-hypertensive agents and/or lipid-lowering agents, were not considered. Fourth, this study did not investigate short-term GV and long-term GV simultaneously and prospectively.

## Conclusions

Visit-to-visit HbA1c variability was inversely related to BRS independently of mean HbA1c in patients with T2DM. Therefore visit-to-visit HbA1c variability might be a marker of reduced BRS in T2DM. Future studies are awaited to focus on the pathophysiology of CAN assessed by BRS.

## Abbreviations

BRS: baroreflex sensitivity
CAN: cardiovascular autonomic neuropathy
CGM: continuous glucose monitoring
GV: glycemic variability
T2DM: type 2 diabetes mellitus
